# Genetic variation and geographic distribution of *Leishmania orientalis* and *Leishmania martiniquensis* among *Leishmania*/HIV co-infection in Thailand

**DOI:** 10.1038/s41598-023-50604-4

**Published:** 2023-12-28

**Authors:** Toon Ruang-areerate, Panthita Ruang-areerate, Jipada Manomat, Tawee Naaglor, Phunlerd Piyaraj, Mathirut Mungthin, Saovanee Leelayoova, Suradej Siripattanapipong

**Affiliations:** 1 Department of Parasitology, Phramongkutklao College of Medicine, Bangkok, 10400 Thailand; 2 BIOTEC, National Science and Technology Development Agency (NSTDA), Pathum Thani, 12120 Thailand; 3 Department of Microbiology, Faculty of Science, Mahidol University, Bangkok, 10400 Thailand

**Keywords:** Phylogenetics, Population genetics, Parasite evolution, Parasitic infection

## Abstract

Since 1999, the number of asymptomatic leishmaniasis cases has increased continuously in Thailand, particularly among patients with HIV who are prone to develop symptoms of cutaneous and visceral leishmaniasis further. The asymptomatic infection could play a key role in *Leishmania* transmission and distribution. Understanding population structure and phylogeographic patterns could be crucially needed to develop effective diagnoses and appropriate guidelines for therapy. In this study, genetic variation and geographic distribution of the *Leishmania*/HIV co-infected population were investigated in endemic northern and southern Thailand. Interestingly, *Leishmania orientalis* was common and predominant in these two regions with common regional haplotype distribution but not for the others. Recent population expansion was estimated, probably due to the movement and migration of asymptomatic individuals; therefore, the transmission and prevalence of *Leishmania* infection could be underestimated. These findings of imbalanced population structure and phylogeographic distribution patterns provide valuable, insightful population structure and geographic distribution of *Leishmania*/HIV co-infection to empower prevention and control of transmission and expansion of asymptomatic leishmaniasis.

## Introduction

Leishmaniasis is one of the neglected tropical diseases related to poverty. Approximately 1.3 million people are infected yearly^1^. The disease is caused by flagellate protozoan parasites in the genus *Leishmania*, transmitted by phlebotomine sandflies in tropical and subtropical regions^2^. A small fraction develops the disease, and 20,000–30,000 cases eventually result in death. The three primary clinical forms include visceral leishmaniasis (VL), cutaneous leishmaniasis (CL), and mucocutaneous leishmaniasis (MCL), with different manifestations ranging from self-healing (CL) to potentially fatal outcomes (VL) so that new cases of VL occur 0.2–0.4 million annually. Currently, at least 18 different *Leishmania* species are pathogenic for humans^3^. A decline in host immune response from HIV transmission probably influences leishmaniasis transmission. Co-infection of leishmaniasis and human immunodeficiency virus (HIV) results in a risk of treatment failure, high relapse, and a high mortality rate^4,5^. Thus, *Leishmania*/HIV co-infection constitutes a significant global public health problem^4,6^.

In Thailand, the number of *Leishmania*/HIV co-infected cases has continuously increased among patients with HIV/AIDS in northern and southern regions due to the emergence of CL and VL caused by *L. (Mundinia) martiniquensis* and *L. (Mundinia) orientalis*^4,7^. An autochthonous VL case was first reported in a 3-year-old girl in 1999 residing in a southern province^8^. In 2012, the first *L. orientalis* was identified in a patient with HIV in Trang province, southern Thailand^9^. Since then, *L. martiniquensis* and *L. orientalis* have been occasionally reported in immunocompetent and immunocompromised patients, predominantly in southern and northern Thailand. Of these, approximately 40% were patients with HIV/AIDS^4,5^. Until now, these two species are the most commonly reported in Thailand^7^.

Asymptomatic *Leishmania* infection could play an important role in disease transmission; however, the reason *Leishmania* distribution was predominantly endemic in northern and southern Thailand requires further study. Low viral load (CD4^+^ levels < 500 cells/µL) and residing in stilt houses were described to be associated with *Leishmania* infection among Thai patients with HIV independently^4^. However, population genetic structure and subtype distribution are poorly understood, which could be responsible for the heterogeneity patterns of disease distribution and promote lineages and/or genetically structured populations restricted to some areas of Thailand. The exchange of genetic material between *Leishmania* populations producing intra- and interspecific hybrids may alter population structure and, thus, complicate epidemiologic studies at the molecular level^10–13^, which is important in effectively diagnosing and providing suitable guidelines for the treatment management of asymptomatic and symptomatic leishmaniasis. Several molecular markers, e.g., nuclear and kinetoplast genes, microsatellites, and internal transcribed spacer 1 (ITS1) have been used to evaluate the genetic diversity of *Leishmania* spp.^10,14–17^. Currently, polymerase chain reaction (PCR) based on the ITS1 has been recommended, comparatively to the direct agglutination test (DAT) assay, to detect *Leishmania* among patients with HIV due to the high sensitivity of *Leishmania* DNA detection^4,18^. An alternative method in addition to traditional PCR detection, the simplified and affordable LAMP assay has been developed to screen and quantify *Leishmania* DNA without the requirement of complex instruments that allowed rapid interpretation and were reliably practical to deliver to end users for field diagnostics in healthcare services^19–22^.

The geographic distribution and population genetic structure of *Leishmania* spp. remains limited, particularly among asymptomatic patients with HIV in Thailand. Based on ITS1 sequences, *Leishmania tropica* showed a complex phylogeographic pattern with dominated haplotypes that were widespread and found across endemic countries, whereas others were restricted to some particular regions^10^. This study assessed population genetic variation and phylogeographic distribution patterns among *Leishmania* species among Thai patients with HIV in endemic northern and southern Thailand. The findings give valuable insights into the dynamics of asymptomatic infection and *Leishmania*/HIV co-infection that could promote an effective diagnosis and proper guidelines for therapy to control leishmaniasis in Thailand.

## Results

### Geographic distribution of isolated population

**Figure 1:**
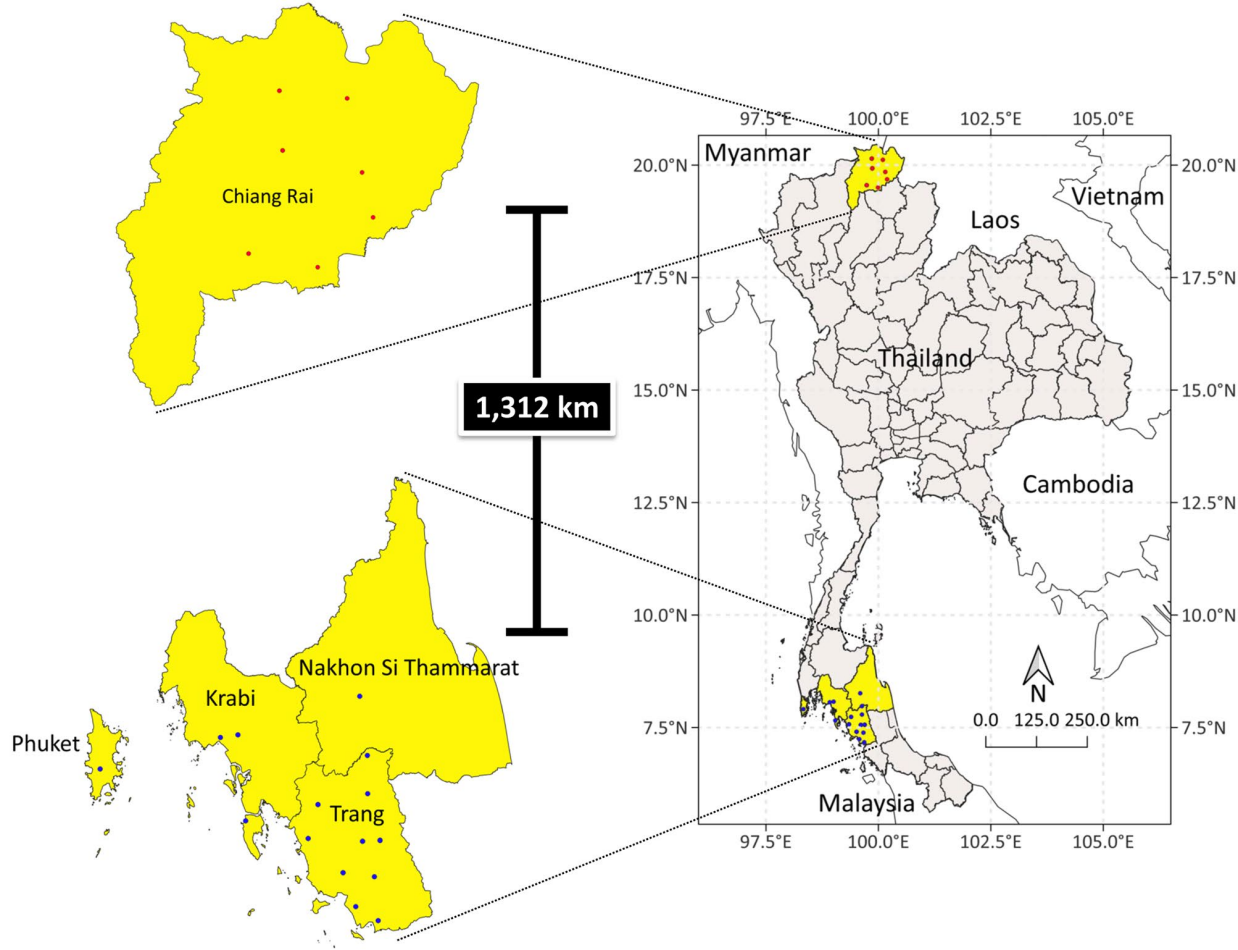
Geographic distribution of 65 *Leishmania* spp. isolates from patients with HIV in 2 endemic regions, northern and southern Thailand. The red circles show isolates in 7 districts of Chiang Rai province in the northern region. The blue circles show isolates in 15 districts of 5 provinces in the southern region (Krabi, Nakhon Si Thammarat, Phuket, Satun, Sonkhla and Trang). The map was created using QGIS Version 3.26.3 (http://www.qgis.org).

A total of 69 *Leishmania* isolates from two endemic regions of Thailand, North (*n* = 15) and South (*n* = 54), were used and genetically characterized using the ITS1 region of the rRNA gene. Four *Leishmania* species and one species complex were shown to associate with HIV patients residing in southern Thailand, including ten districts of Trang province, three districts of Krabi, and one district of Nakhon Si Thammarat and Phuket, respectively (Table S1). Unlikely, *L. orientalis* was predominant and widely distributed across seven districts of Chiang Rai Province in the northern region. In contrast, only a single isolate was genetically identified as *L. martiniquensis* in the Phan (N1D2) district, approximately 1300 km from the endemic southern region. The district locations of all isolates (*n* = 65, except four isolates with unknown locations) were presented in Fig. 1. Each isolate's location code and coordinate were assigned and shown in Table S1.

### Phylogenetic analysis

**Figure 2:**
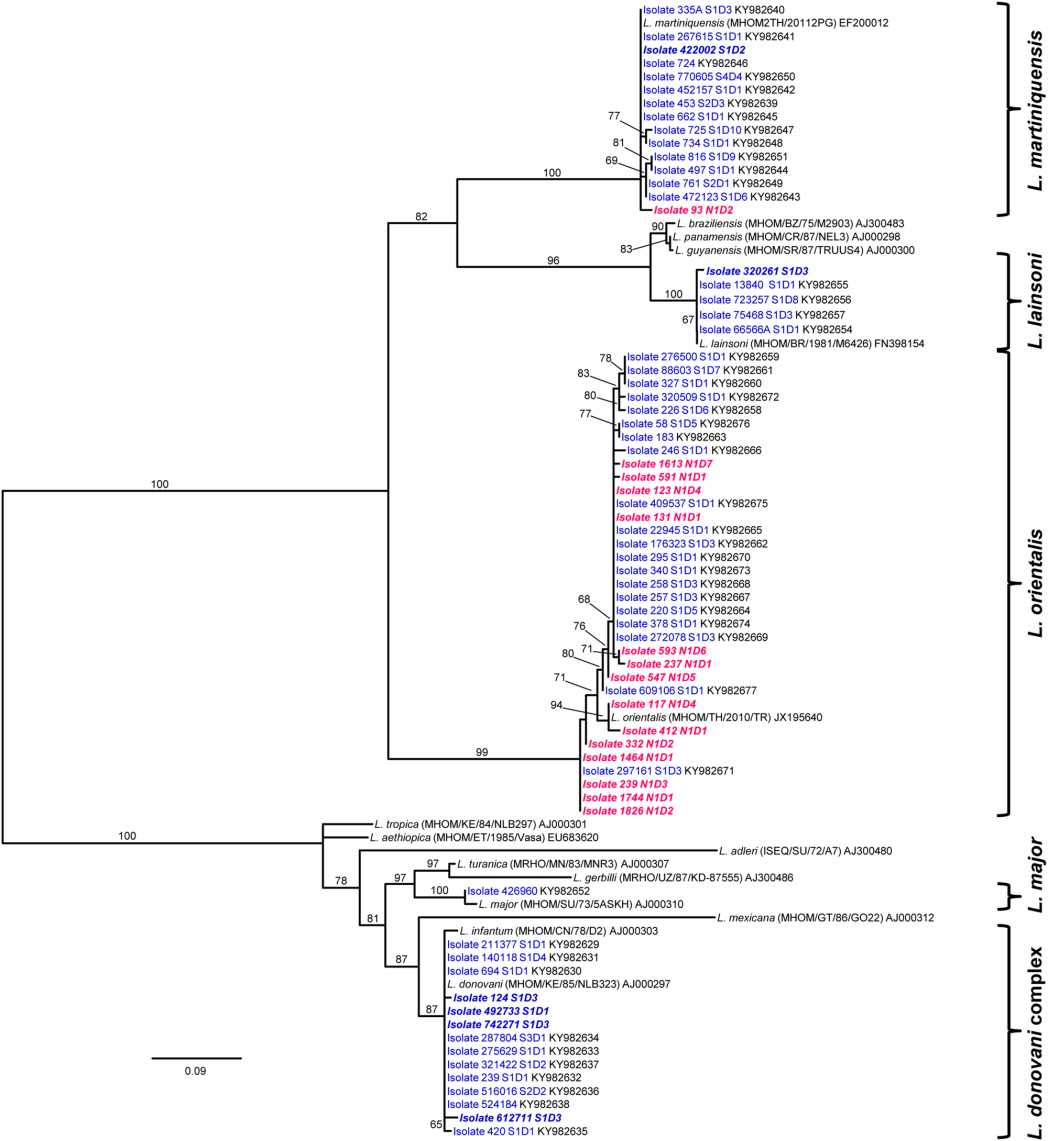
Randomized accelerated maximum likelihood (RAxML) tree of *Leishmania* species was based on the internal transcribed spacer 1 (ITS1) region of the ribosomal RNA (rRNA) gene sequences. The tree was unrooted. Bootstrap values (50,000 replicates) are presented as percentages above the individual branches in which branches with values < 50% are not shown. *Leishmania* isolates from southern Thailand are indicated in blue and northern Thailand in red. Italic boldface denotes for isolates in this study, while isolates from Manomat et al.^4^ are presented in plain text.

A phylogenetic tree of *Leishmania* species based on the ITS1 region of the rRNA gene was constructed using 69 DNA sequences of isolates from endemic northern and southern Thailand (21 in this study and 48 conducted by Manomat et al.^4^), and 15 *Leishmania* references retrieved from GenBank. Figure 2 illustrates an unrooted RAxML tree of *Leishmania* isolates in asymptomatic co-infected patients with HIV in Thailand. The tree topology demonstrates that *Leishmania* infection in HIV patients could be classified in four species and one species complex including *L. orientalis* (49.3%, *n* = 34), *L. martiniquensis* (21.7%, *n* = 15), *L. lainsoni* (7.2%, *n* = 5) and *L. major* (1.5%, *n* = 1) with strongly supported bootstrap values at 99 to 100% except for *L. donovani* complex (20.3%, *n* = 14; as *L. donovani* and *L. infantum*), bootstrap value = 87%. *Leishmania* isolates in 15 districts of three southern provinces (78.3%, *n* = 54) showed clusters in all species and species complex assemblages. In contrast, the isolates of northern Thailand (21.7%, *n* = 15) in 7 districts were found to be *L. orientalis* and *L. martiniquensis*. Interestingly, *L. orientalis* (49.3%, *n* = 34) was predominant and commonly infected among patients living in the north and south of Thailand (n = 14 and 20, respectively); otherwise, the other species were comparatively found to be geographically associated with the southern region.

### Genetic and haplotype diversities

Among three *Leishmania* species and one species complex, the highest haplotype diversity (Hd) and nucleotide diversity (Pi; π) were 0.850 ± 0.053 and 0.009 ± 0.001 in *L. orientalis*, respectively, based on the ITS1 region of the rRNA gene in which *L. orientalis* in northern Thailand was comparatively higher than south (Hd = 0.923 ± 0.060, Pi(π) = 0.012 ± 0.002 in north; 0.719 ± 0.105, 0.007 ± 0.005 in south, respectively) (Table 1). In the south, haplotype diversity (Hd) of *L. orientalis* and *L. martiniquensis* (0.719 ± 0.105; 0.692 ± 0.115, respectively) were comparatively higher than *L. donovani* complex and *L. lainsoni* (0.295 ± 0.156; 0.400 ± 0.237, respectively). Otherwise, nucleotide diversity (Pi; π) was relatively low for three *Leishmania* species and one species complex ranging between 0.002 and 0.009. In all, the highest number of haplotypes was found within the HIV-coinfected population of *L. orientalis* (haplotype = 16; *n* = 33) but less for *L. martiniquensis* (haplotype = 5; *n* = 14), *L. donovani* complex (haplotype = 3; *n* = 13) and *L. lainsoni* (haplotype = 2; *n* = 5) (Table 2).

**Table 1:** Genetic diversity and neutrality test of the population of *Leishmania* species and species complex in the patients with HIV in northern and southern Thailand using ITS1 region of the rRNA gene sequences.

Population	Sample size (*n*)	Haplotype	No. of polymorphic sites	Genetic diversity^a^		Result for neutrality tests^b^	
				Hd ± SD	Pi (π) ± SD	Tajima's *D*	Fu's *Fs*
*L. orientalis*							
North	14	10	9	0.923 ± 0.060	0.012 ± 0.002	0.305	− 4.505
South	19	8	10	0.719 ± 0.105	0.007 ± 0.005	− 1.444	− 2.944
Total	33	16	15	0.850 ± 0.053	0.009 ± 0.001	− 0.991	− 8.726
*L. martiniquensis*							
North	1	1	–	–	–	–	–
South	13	4	2	0.692 ± 0.115	0.003 ± 0.002	1.214	− 0.687
Total	14	5	4	0.736 ± 0.107	0.004 ± 0.003	− 0.243	− 1.250
*L. donovani* complex	13	3	3	0.295 ± 0.156	0.002 ± 0.001	− 1.652	− 0.689
*L. lainsoni*	5	2	3	0.400 ± 0.237	0.005 ± 0.003	− 1.048	1.688

^a^Hd, haplotype diversity; Pi (π), nucleotide diversity.

^b^No values were significant, at a *P*-value of > 0.05.

**Table 2:** Coordinates and haplotype frequencies of four *Leishmania* species and one species complex co-infected in patients with HIV from northern and southern regions of Thailand.

Species	Region, Province, District	Coordinates (Lat., Long.)	*n*	Haplotype; H (Frequency)	Location code
*L. orientalis*	North (N), Chiang Rai (1), Mueang (D1)	19°55′44.6″N 99°51′51.9″E	6	H2_Lo_(1), H6_Lo_(2), H9_Lo_(1), H10_Lo_(1), H11_Lo_(1)	N1D1
	North (N), Chiang Rai (1), Phan (D2)	19°33′14.2″N 99°44′25.7″E	2	H6_Lo_(1), H12_Lo_(1)	N1D2
	North (N), Chiang Rai (1), Thoeng (D3)	19°41′08.7″N 100°11′37.3″E	1	H6_Lo_(1)	N1D3
	North (N), Chiang Rai (1), Mae Chan (D4)	20°08′46.1″N 99°51′09.6″E	2	H2_Lo_(1), H13_Lo_(1)	N1D4
	North (N), Chiang Rai (1), Padad (D5)	19°30′15.4″N 99°59′31.6″E	1	H15_Lo_(1)	N1D5
	North (N), Chiang Rai (1), Phaya Mengrai (D6	19°50′57.0″N 100°09′12.8″E	1	H14_Lo_(1)	N1D6
	North (N), Chiang Rai (1), Doi Luang (D7)	20°07′06.4″N 100°05′57.2″E	1	H16_Lo_(1)	N1D7
	South (S), Trang (1), Mueang (D1)	7°33′28.7″N 99°36′35.1″E	10	H1_Lo_(2), H2_Lo_(4), H3_Lo_(1), H4_Lo_(1), H5_Lo_(1), H6_Lo_(1)	S1D1
	South (S), Trang (1), Kantang (D3)	7°24′19.9″N 99°30′53.9″E	5	H2_Lo_(4), H6_Lo_(1)	S1D3
	South (S), Trang (1), SiKao (D5)	7°34′18.5″N 99°20′43.1″E	2	H2_Lo_(1), H7_Lo_(1)	S1D5
	South (S), Trang (1), Nayong (D6)	7°33′42.5″N 99°41′41.8″E	1	H8_Lo_(1)	S1D6
	South (S), Trang (1), Yantakao D7)	7°23′09.1″N 99°40′02.2″E	1	H1_Lo_(1)	S1D7
	South (S)	N/A	1	N/A	N/A
Subtotal			34		
*L. martiniquensis*	North (N), Chiang Rai (1), Phan (D2)	19°33′14.2″N 99°44′25.7″E	1	H5_Lm_(1)	N1D2
	South (S), Trang (1), Mueang (D1)	7°33′28.7″N 99°36′35.1"E	5	H1_Lm_(3), H3_Lm_(1), H4_Lm_(1)	S1D1
	South (S), Trang (1), Wang Wiset (D2)	7°44′10.7″N 99°23′34.5″E	1	H1_Lm_(1)	S1D2
	South (S), Trang (1), Kantang (D3)	7°24′19.9″N 99°30′53.9″E	1	H2_Lm_(1)	S1D3
	South (S), Trang (1), Nayong (D6)	7°33′42.5″N 99°41′41.8″E	1	H3_Lm_(1)	S1D6
	South (S), Trang (1), Hat Samran (D9)	7°14′26.6″N 99°34′36.2″E	1	H3_Lm_(1)	S1D9
	South (S), Trang (1), Ratsada (D10)	7°58′29.3″N 99°37′59.5″E	1	H4_Lm_(1)	S1D10
	South (S), Krabi (2), Mueang (D1)	8°03′44.1″N 98°55′06.6″E	1	H2_Lm_(1)	S2D1
	South (S), Krabi (2), Nuea Khlong (D3)	8°04′29.6″N 99°00′13.2″E	1	H1_Lm_(1)	S2D3
	South (S), Phuket (4) Kathu (D1)	7°54′32.7″N 98°20′00.4″E	1	H1_Lm_(1)	S4D1
	South (S)	N/A	1	N/A	N/A
Subtotal			15		
*L. donovani *complex	South (S), Trang (1), Mueang (D1)	7°33′28.7″N 99°36′35.1″E	6	H1_Ld_(5), H2_Ld_(1)	S1D1
	South (S), Trang (1), Wang Wiset (D2)	7°44′10.7″N 99°23′34.5″E	1	H1_Ld_(1)	S1D2
	South (S), Trang (1), Kantang (D3)	7°24′19.9**″**N 99°30′53.9**″**E	3	H1_Ld_(2), H3_Ld_(1)	S1D3
	South (S), Trang (1), Nayong Nuea (D4)	7°33′51.5″N 99°41′41.7″E	1	H1_Ld_(1)	S1D4
	South (S), Krabi (2), Koh Lanta (D2)	7°39′26.5″N 99°02′28.6″E	1	H1_Ld_(1)	S2D2
	South (S), Nakhon Si Thammarat (3), Na Bon (D1)	8°15′44.0″N 99°35′46.1″E	1	H1_Ld_(1)	S3D1
	South (S)	N/A	1	N/A	N/A
Subtotal			14		
*L. lainsoni*	South (S), Trang (1), Mueang (D1)	7°33′28.7″N 99°36′35.1″E	2	H1_Ll_(1)	S1D1
	South (S), Trang (1), Kantang (D3)	7°24′19.9″N 99°30′53.9″E	2	H1_Ll_(1), H2_Ll_(1)	S1D3
	South (S), Trang (1), Palien (D8)	7°10′21.8″N 99°41′08.9″E	1	H1_Ll_(1)	S1D8
Subtotal			5		
*L. major*	South (S)	N/A	1	N/A	N/A
Subtotal			1		
Total			**69**		

N/A: not applicable.

**Figure 3:**
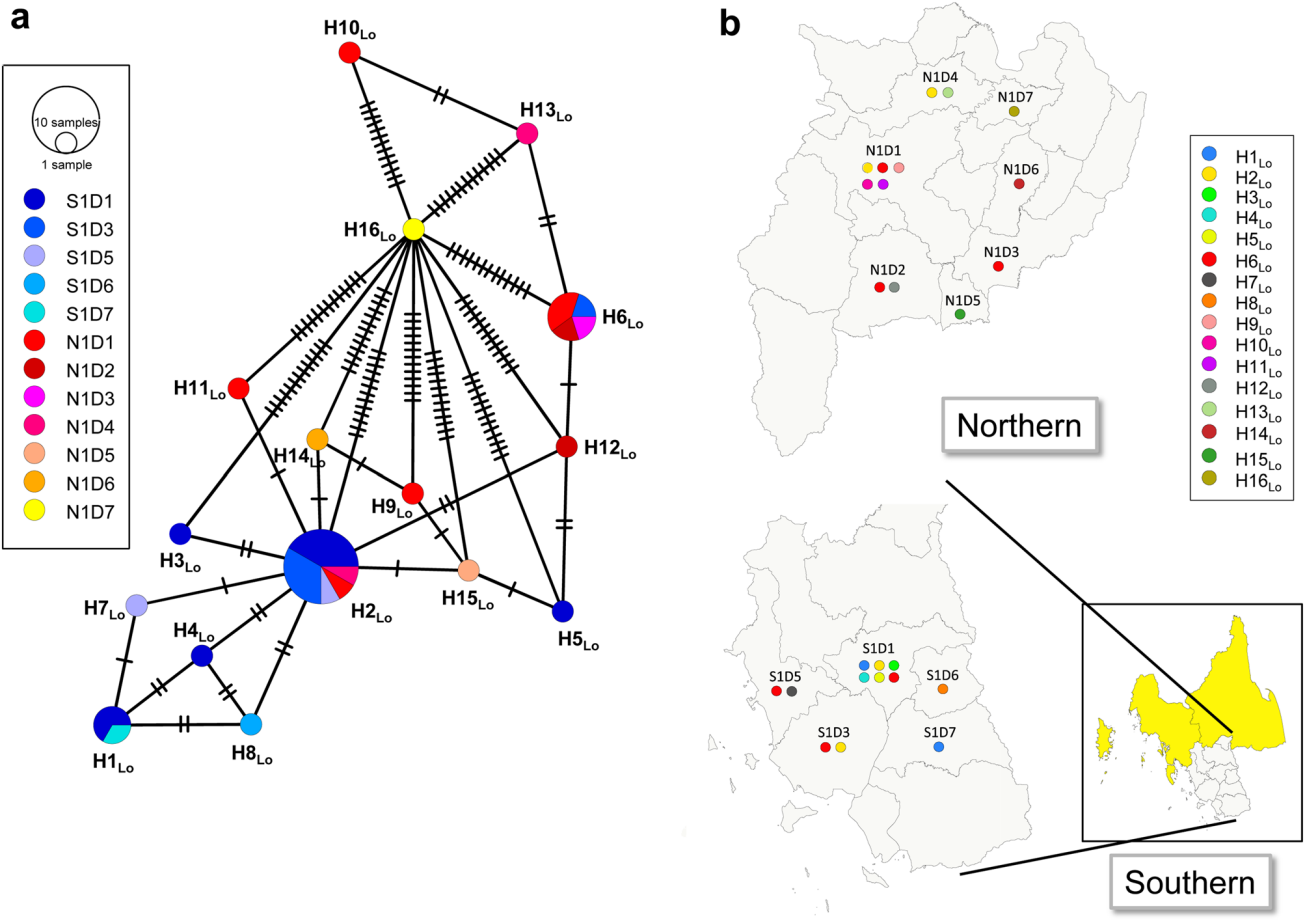
Haplotype network and geographic distribution of *L. orientalis* haplotypes in endemic northern and southern regions of Thailand. (**a**) Minimum spanning network inferred from ITS1 region of the rRNA gene sequences of *L. orientalis* (Lo) in 2 endemic regions, Thailand. Circles represent common haplotypes (H). Circle size is proportionally relative to the haplotype frequency where the color scheme represents the collecting district location (D) in the north (N); hot color, and the south (S); cold color. Branch lengths are proportional to a single-nucleotide change indicated by the number of crossing bars. (**b**) Geographic distribution of *L. orientalis* (Lo) haplotypes in northern and southern provinces of Thailand. Haplotypes of isolates in each collecting district location (D) in the north (N) and the south (S) are shown in circles. The map was created using QGIS Version 3.26.3 (http://www.qgis.org).

**Figure 4:**
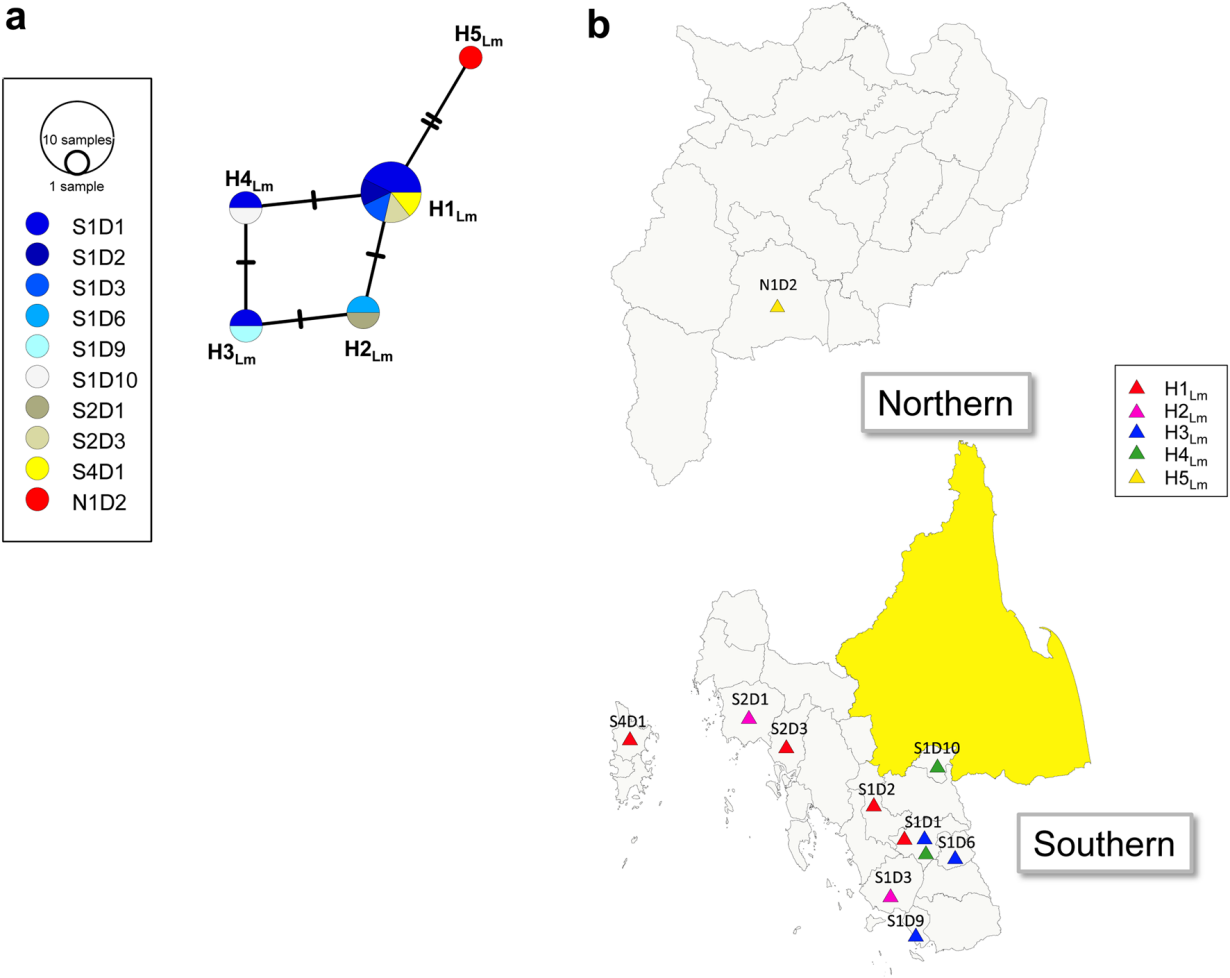
Haplotype network and geographic distribution of *L. martiniquensis* haplotypes in endemic northern and southern regions of Thailand. (**a**) Minimum spanning network inferred from ITS1 region of the rRNA gene sequences of *L. martiniquensis* (Lm) in 2 endemic regions, Thailand. Circles represent common haplotype (H). Circle size is proportionally relative to the haplotype frequency that the color scheme represents the collecting district location (D) in the north (N); hot color, and the south (S); cold color. Branch lengths are proportional to a single-nucleotide change indicated by the number of crossing bars. (**b**) Geographic distribution of *L. martiniquensis* (Lm) haplotypes in northern and southern provinces of Thailand. Haplotypes of isolates in each collecting district location (D) in the north (N) and the south (S) are shown in triangles. The map was created using QGIS Version 3.26.3 (http://www.qgis.org).

The haplotype network demonstrated a complex haplotype structure of *L. orientalis* (Fig. 3a) but not for other *Leishmania* species. The numbers of haplotypes were low, ranging from 2 to 5 haplotypes (Figs. 4a, S1a and c) and geographically distributed mainly in southern Thailand (Figs. 4b, S1b and d). Additionally, *L. orientalis* was commonly found in both endemic northern and southern regions (Fig. 3b). The common haplotypes were H2_Lo_ and H6_Lo_ for *L. orientalis* (Fig. 3a), H1_Lm_ for *L. martiniquensis* (Fig. 4a), H1_Ld_ for *L. donovani* complex (Fig. S1a) and H1_Ll_ for *L. lainsoni* (Fig. S1c), respectively. In the south, H2_Lo_ was a dominant haplotype among HIV patients, whereas in the northern region, it was H6_Lo_. Nonetheless, H2_Lo_ and H6_Lo_ were shared between isolates of asymptomatic patients with HIV from both regions of Thailand, with closely related singletons of isolates in each region except H16_Lo_. The overall Tajima's *D* and Fu's *Fs* tests were negative for *L. orientalis* (*D* =  − 0.991; *Fs* =  − 8.726, respectively), *L. martiniquensis* (− 0.243; − 1.250, respectively) and *L. donovani* complex (− 1.652; − 0.689, respectively) indicating a bias toward rare alleles, being a signature of recent population expansion, causing possible recent population growth (Table 1). However, the result of *L. lainsoni* isolates conflicted with *Fs* of 1.688 but *D* of − 1.048. The *P*-values of *D* and *Fs* tests accepted the null hypothesis for all populations, suggesting a neutral population.

## Discussion

This study showed that *L. orientalis* of asymptomatic patients with HIV was predominant and widely geographically distributed in northern and southern Thailand. The others were primarily associated with the south region, including *L. martiniquensis*, *L. lainsoni,* and *L. donovani* complex, except *L. major*, which was only a single isolate reported by Manomat et al.^4^. In Thailand, three known causative leishmaniasis species are *L. orientalis*, *L. martiniquensis,* and *L. donovani* complex^23^. Manomat et al.^4^ have recently characterized and reported the infection of *L. lainsoni* among HIV patients in 2017. Interestingly, this study has also found and characterized *L. lainsoni* (Isolate 320261 S1D3) from a new *Leishmania*/HIV co-infected case in the Kantang district of Trang province, suggesting the prevalence of this species in the south of Thailand. Thus, the mixed species of *Leishmania* were commonly found in the southern region that shared the same HIV patient population. Regarding the ~ 1300 km distance between northern and southern Thailand, the distribution of *L. orientalis* infection could be common, resulting in a significant public health problem of leishmaniasis in Thailand than others. However, the prevalence of *Leishmania* infection varied between the two regions, with 25.1% in the south and 7.1% in the north of Thailand^4,24^.

Based on the ITS1 region of the rRNA gene sequences, the RAxML tree shows a tree topology similar to Manomat et al.^4^, who demonstrated phylogenetic relationships of *Leishmania* infection in this southern population. Four species, including *L. orientalis*, *L. martiniquensis*, *L. lainsoni,* and *L. major*, and one species complex (*L. donovani* complex) have been classified with highly supported bootstrap values (≥ 99%) in node divergences of four species except a node of *L. donovani* complex (87%), in agreement with previous work^4^. In Thailand, leishmaniasis is an emerging disease with unknown incidence or prevalence that new cases have been further described, undoubtedly reflecting a much higher prevalence of undiagnosed and asymptomatic cases^23^, particularly in patients with HIV^4^. Jariyapan et al.^23^ provided an updated taxonomical identification of all known *Leishmania* species in Thailand into *L. orientalis*, *L. martiniquensis*, and *L. infantum* (*L. donovani* complex). However, *L. orientalis* showed a complex phylogeographic pattern broadly found in distant differences (1312 km) between northern and southern regions, whereas the other species were mainly restricted to southern Thailand.

Of these *Leishmania* species, a complex haplotype network was observed in *L. orientalis*, including 16 haplotypes, but not for the others that showed diversely less than five haplotypes. Interestingly, the haplotype network of *L. orientalis* has demonstrated the regionally restricted geographic distribution of haplotypes in the north and south, where the two major common haplotypes were H6_Lo_ for the north and H2_Lo_ for the south. In contrast to *L. orientalis*, the haplotypes of other *Leishmania* species were restricted and geographically distributed in the southern region, except for H5_Lm_ of *L. martiniquensis*, which was found in the northern region. The mixed haplotype distribution of *Leishmania* species and species complex was observed in many district foci; therefore, coexistence and competition could occur and provoke selective pressure on some haplotypes and species dominating within the regional population. Unlike other species, a large number of closely related haplotypes of *L. orientalis* was estimated probably due to high probabilities of the recombination rate within populations; nevertheless, partially restricting isolation of subregional geographic distribution could promote an occurrence of intraspecific recombination within each location and result in common regional haplotypes. *Leishmania tropica* showed a complex phylogeographic pattern in some dominated haplotypes widespread across endemic countries, whereas others were restricted to particular regions based on the ITS1 sequences^10^.

*Leishmania orientalis* had high genetic diversity (Hd), especially in northern Thailand, indicating that the population had higher probabilities of a recombination rate than the others, leading to a large number of closely related haplotypes, whereas low nucleotide diversity (Pi; π) implied that minor differences between haplotypes were found within a population^25^. Otherwise, genetic diversity and nucleotide diversity were relatively low for other species in both regions of Thailand. These diversity indices can be described by the recent expansion of the species and gene flow between the regions. A sufficient gene flow may slow or prevent the geographic differentiation between populations, resulting in subpopulation structure over a large area^25,26^. Tajima's *D* and Fu's *Fs* tests indicated that the populations of *Leishmania* species and species complex in asymptomatic patients with HIV were under bias toward rare alleles^27^ and possible recent population growth^28^ except for *L. lainsoni,* which was conflicted with positive *Fs* value. The overall negative values of the two tests demonstrated an excess of rare mutations in populations that resulted in recent population expansion. Thus, these populations were under evolving mutations randomly or neutral populations; however, further study is needed regarding the limited population size in each *Leishmania* species and species complex^29^. Additionally, evaluating other neutral nuclear DNA markers could estimate a better understanding of the neutral population structure^25^.

In northern and southern Thailand, *Leishmania*/HIV co-infected cases have continuously increased among patients with HIV/AIDS^4,7^. In contrast, the two most common species, *L. martiniquensis* and *L. orientalis* have been occasionally reported in immunocompetent and immunocompromised patients^4,5,7^. Most infections caused by these two species were asymptomatic. However, symptomatic CL and VL were reported in HIV-infected patients with a CD4 count of less than 200 cells/mm^3^ who were infected by *L. martiniquensis* and *L. orientalis* in Thailand^4,5,7^. This study estimated recent population expansion and indicated that *Leishmania* species are restricted and predominant in some regions. Asymptomatic infection in immunocompetent and immunocompromised patients may crucially be a key factor in the transmission and expansion of *Leishmania*, particularly in *L. orientalis*, in different regions of Thailand due to the limitation of effective diagnosis and suitable guidelines therapy to manage and control the movement and migration of infected population. In addition to the substantial barrier in these study sites, the rapid growth of frequent air travel might spread and transfer the parasites between regions in both directions.

Further study in genetic variation of large infected populations is required to estimate the proper population structure of dominated and common regional haplotypes, and geographic distribution. Studies using other highly polymorphic markers, such as kDNA and microsatellites, could additionally identify genetic variation at the strain level and population structure in the future. Thus, identifying vectors and reservoir hosts further needs to limit and control *Leishmania* transmission within the population.

## Conclusion

In conclusion, we have demonstrated genetic variation and geographic distribution of *Leishmania*/HIV co-infection in Thailand, where *L. orientalis* was predominant in northern and southern regions but not in the others. Regarding the haplotype network, common regional haplotypes were observed in *L. orientalis*. Recent population expansion has been estimated, suggesting that the movement and migration of asymptomatic infection could play a vital role in the transmission and expansion of *Leishmania* in two regions, and the prevalence of *Leishmania* infection could be underestimated. However, the DNA sequences of *Leishmania* species represented the population genetic structure and geographic distribution of *Leishmania* spp. in asymptomatic patients with HIV. Further study of *Leishmania* infection among immunocompetent patients is required to reveal the actual population genetic structure and geographic distribution of *Leishmania* spp. in Thailand. The findings of the imbalanced population structure and phylogeographic distribution pattern give valuable insights into the dynamics of asymptomatic *Leishmania*/HIV co-infection and public health awareness that an effective diagnosis and appropriate guidelines for therapy are required to prevent and control the transmission and expansion of *Leishmania* infection.

## Materials and methods

### Ethics statement

Blood samples were collected for diagnostic purposes. All subjects, aged > 18 years were informed and enrolled in the study. Written informed consent was received from all subjects before blood samples were collected. Samples were coded and anonymized before specimen processing and analysis. All methods were carried out according to relevant guidelines and regulations. The experiment was reviewed and approved by the Ethics Committee of the Royal Thai Army Medical Department (IRBRTA 0572/2564, IRBRTA 811/2565 and IRBRTA 812/2565).

### Study population and specimen collection

A study of population genetic structure and geographic distribution of *Leishmania* species among co-infected Thai patients with HIV was conducted in endemic southern and northern regions, including 15 districts of three southern provinces and seven districts of one northern province of Thailand (Fig. 1). The *Leishmania* in northern Thailand (Chiang Rai province; *n* = 15) and southern Thailand (Trang, Krabi, and Nakhon Si Thammarat provinces; *n* = 6) from co-infected patients with *Leishmania*/HIV during 2021, and 48 *Leishmania* isolates from Trang in 2017^4^ were characterized and used in this study (Table 2). Eight milliliters (mL) of EDTA anticoagulated blood were collected from subjects > 18 years old attending the HIV Clinic at Trang Hospital, Trang province, and the HIV Clinic at Chiangrai Prachanukroh Hospital, Chiang Rai province, during a 6-month follow-up period to receive antiretroviral therapy (ART). Plasma and buffy coat were separated from the whole blood specimen by centrifuging at 900 × *g* for 10 min and kept at − 20 °C for further DNA extraction.

### DNA extraction and PCR amplification

The total DNA of buffy coat samples was extracted using the Geneaid™ DNA Isolation Kit (blood) (New Taipei, Taiwan) according to manufacturer protocols and stored at − 20 °C until used. The ITS1 region of the ribosomal RNA (rRNA) gene of *Leishmania* was amplified based on nested PCR described by Manomat et al.^4^ using a FlexCycler2 Thermocycler (Analytik Jena, Jena, Germany) with promastigotes' DNA of *L. martiniquensis* (WHOM/TH/2011/PG) as a positive control. PCR products were detected by electrophoresis in 1.5% agarose gel stained with SYBR™ Safe DNA Gel Stain (Thermo Fisher Scientific, Waltham, MA, USA) and visualized using a Molecular Imager® Gel Doc™ XR + System with Imager Lab^TM^3.0 (Bio-Rad, Hercules, CA, USA).

### DNA sequencing and phylogenetic analysis

The positive *Leishmania*/HIV samples were purified and sent to Bionics Co. Ltd. (Seoul, South Korea) for direct sequencing. The chromatograms of each sequence were validated and manually edited using BioEdit, Version 7.2.5^30^. To perform phylogenetic analysis, the 69 ITS1 gene sequences (*n* = 69) obtained in this study and Manomat et al.^4^ were multiple aligned with a set of 15 reference sequences of *Leishmania* species retrieved from the GenBank database using ClustalW in BioEdit. The phylogenetic tree was constructed based on Randomized Axelerated Maximum Likelihood (RAxML), Version 7.4.2, with a GTR matrix (GTR + Γ model)^31^ using RaxmlGUI, Version 1.3^32^. Clade stability was evaluated using 50,000 replicates of RAxML bootstrap values. The best ML tree is estimated to provide an evolutionary tree for understanding biodiversity. RAxML was chosen because it had the fastest ML analysis on large datasets^31^. Therefore, the ML tree was used to obtain evolutionary relationships among our accessions. All sequences were deposited to GenBank under Accession No. OR135533 to OR135553 (Table S1).

### Population genetic analysis

To determine the genetic variation of the ITS1 region of the rRNA gene among 69 isolates of *Leishmania* species and species complex (21 in this study and 48 conducted by Manomat et al.^4^), haplotype diversity (Hd) and nucleotide diversity (Pi; π) were calculated using the DnaSP, Version 6.0^33^. A haplotype network was constructed to investigate relationships among haplotypes using a minimum-spanning haplotype network with PopART, Version 1.7^34^. A selective neutrality test was analyzed to determine genetic hitchhiking, population expansion, selective sweep and bottleneck using the statistical significance of Tajima's *D* and Fu's *Fs* tests at 95% intervals (*P*-value < 0.05)^27,28^.

### Supplementary Information

Supplementary Information.

## Data Availability

The datasets generated during the current study are available from the corresponding author upon reasonable request.
