# Supplementary material for: Genetic variation and geographic distribution of Leishmania orientalis and Leishmania martiniquensis among Leishmania/HIV co-infection in Thailand
Source: Sci Rep. 2023 Dec 28;13:23094. doi: 10.1038/s41598-023-50604-4 (PMC10754904; doi:10.1038/s41598-023-50604-4)
Supplement: Supplementary file 1 — Supplementary Information. [file 41598_2023_50604_MOESM1_ESM.pdf]

Genetic variation and geographic distribution of *Leishmania orientalis* and *Leishmania martiniquensis* among *Leishmania*/HIV co-infection in Thailand

Toon Ruang-areerate[1*], Panthita Ruang-areerate[2], Jipada Manomat[3], Tawee Naaglor[1], Phunlerd Piyaraj[1], Mathirut Mungthin[1], Saovanee Leelayoova[1] and Suradej Siripattanapipong[3*]

[1]Department of Parasitology, Phramongkutklao College of Medicine, Bangkok 10400 Thailand.
[2]BIOTEC, National Science and Technology Development Agency (NSTDA), Pathum Thani 12120 Thailand.
[3]Department of Microbiology, Faculty of Science, Mahidol University, Bangkok 10400 Thailand.

*Address correspondence to Toon Ruang-areerate, Department of Parasitology, Phramongkutklao College of Medicine, Bangkok 10400 Thailand and Suradej Siripattanapipong, Department of Microbiology, Faculty of Science, Mahidol University, Bangkok 10400 Thailand.
E-mail: youangtr@yahoo.com and suradej.sir@mahidol.ac.th

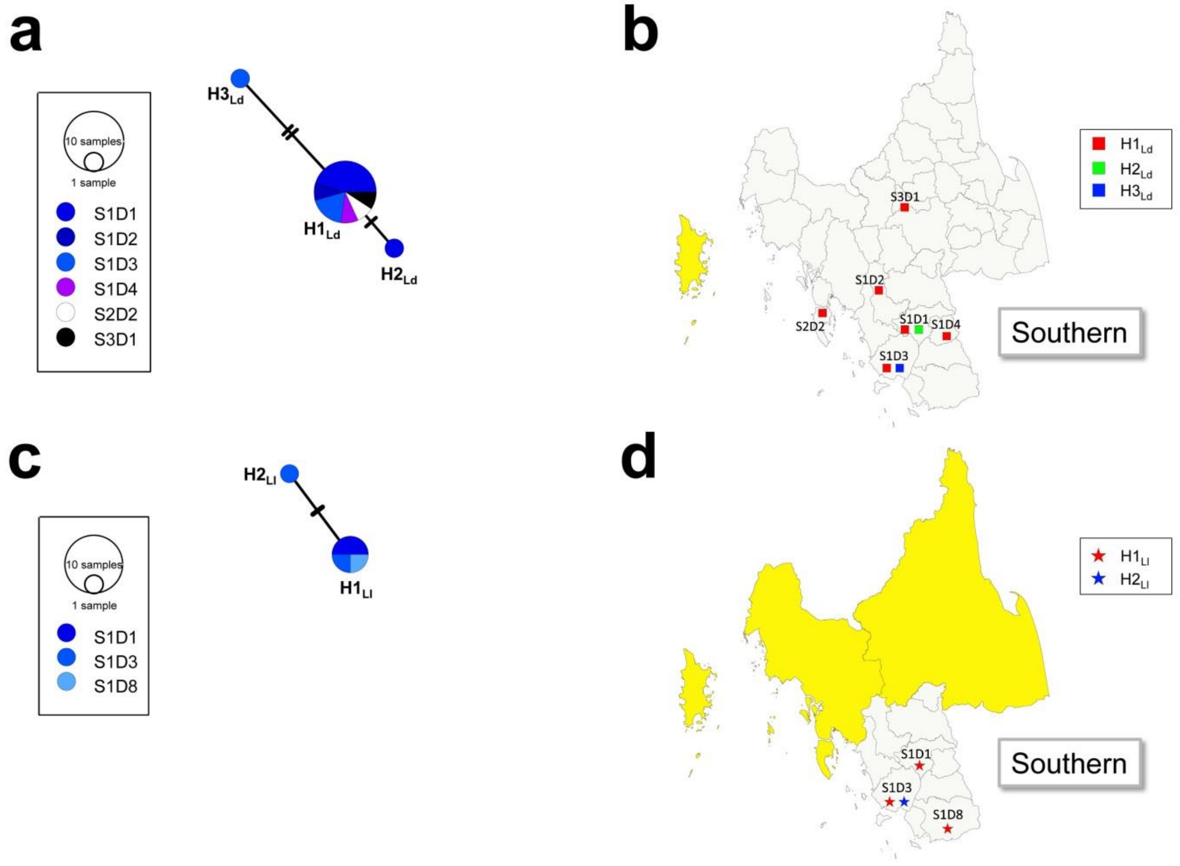

Figure S1. Haplotype network and geographic distribution of *L. donovani* complex and *L. lainsoni* haplotypes in the southern region of Thailand. (a) Minimum spanning network inferred from ITS1 region of the rRNA gene sequences of *L. donovani* complex (Ld) in southern Thailand. Circles represent common haplotypes (H). Circle size is proportionally relative to the haplotype frequency that the cold color scheme represents the collecting district location (D) in south (S). Branch lengths are proportional to a single-nucleotide change indicated by number of crossing bars. (b) Geographic distribution of *L. donovani* complex (Ld) haplotypes in southern provinces of Thailand. Haplotypes of isolates in each collecting district location (D) in south (S) are shown in squares. (c) Minimum spanning network inferred from ITS1 region of the rRNA gene sequences of *L. lainsoni* (Ll) in southern Thailand. Circles represent common haplotypes (H). Circle size is proportionally relative to the haplotype frequency that the cold color scheme

represents the collecting district location (D) in south (S). Branch lengths are proportional to a single-nucleotide change indicated by number of crossing bars. (d) Geographic distribution of *L. lainsoni* (Ll) haplotypes in southern provinces of Thailand. Haplotypes of isolates in each collecting district location (D) in south (S) are shown in stars. The map was created using QGIS Version 3.26.3 (http://www.qgis.org).

Species	Region	Province	District	Isolate name	Location code	Accession no.	Coordinate (lat., long.)	Source
L. orientalis								
	North (N)	Chiang Rai (1)	Mueang (D1)	131	N1D1	OR135533	19°55'44.6"N 99°51'51.9"E	In this study
				237	N1D1	OR135534	19°55'44.6"N 99°51'51.9"E	In this study
				412	N1D1	OR135535	19°55'44.6"N 99°51'51.9"E	In this study
				591	N1D1	OR135536	19°55'44.6"N 99°51'51.9"E	In this study
				1464	N1D1	OR135537	19°55'44.6"N 99°51'51.9"E	In this study
				1744	N1D1	OR135538	19°55'44.6"N 99°51'51.9"E	In this study
			Phan (D2)	332	N1D2	OR135539	19°33'14.2"N 99°44'25.7"E	In this study
				1826	N1D2	OR135540	19°33'14.2"N 99°44'25.7"E	In this study
			Thoeng (D3)	239	N1D3	OR135541	19°41'08.7"N 100°11'37.3"E	In this study
			Mae Chan (D4)	117	N1D4	OR135542	20°08'46.1"N 99°51'09.6"E	In this study
				123	N1D4	OR135543	20°08'46.1"N 99°51'09.6"E	In this study
			Mae Chan (D5)	547	N1D5	OR135544	19°30'15.4"N 99°59'31.6"E	In this study
			Phaya Mengrai (D6)	593	N1D6	OR135545	19°50'57.0"N 100°09'12.8"E	In this study
			Doi Luang (D7)	1613	N1D7	OR135546	20°07'06.4"N 100°05'57.2"E	In this study
	South (S)	Trang (1)	Mueang (D1)	276500	S1D1	KY982659	7°33'28.7"N 99°36'35.1"E	Manomat et al. 2017[4]
				327	S1D1	KY982660	7°33'28.7"N 99°36'35.1"E	Manomat et al. 2017[4]
				22945	S1D1	KY982665	7°33'28.7"N 99°36'35.1"E	Manomat et al. 2017[4]
				246	S1D1	KY982666	7°33'28.7"N 99°36'35.1"E	Manomat et al. 2017[4]
				295	S1D1	KY982670	7°33'28.7"N 99°36'35.1"E	Manomat et al. 2017[4]
				320509	S1D1	KY982672	7°33'28.7"N 99°36'35.1"E	Manomat et al. 2017[4]
				340	S1D1	KY982673	7°33'28.7"N 99°36'35.1"E	Manomat et al. 2017[4]

Species	Region	Province	District	Isolate name	Location code	Accession no.	Coordinate (lat., long.)	Source
				378	S1D1	KY982674	7°33'28.7"N<br>99°36'35.1"E	Manomat et al. 2017[4]
				409537	S1D1	KY982675	7°33'28.7"N<br>99°36'35.1"E	Manomat et al. 2017[4]
				609106	S1D1	KY982677	7°33'28.7"N<br>99°36'35.1"E	Manomat et al. 2017[4]
			Kantang (D3)	176323	S1D3	KY982662	7°24'19.9"N<br>99°30'53.9"E	Manomat et al. 2017[4]
				257	S1D3	KY982667	7°24'19.9"N<br>99°30'53.9"E	Manomat et al. 2017[4]
				258	S1D3	KY982668	7°24'19.9"N<br>99°30'53.9"E	Manomat et al. 2017[4]
				272078	S1D3	KY982669	7°24'19.9"N<br>99°30'53.9"E	Manomat et al. 2017[4]
				297161	S1D3	KY982671	7°24'19.9"N<br>99°30'53.9"E	Manomat et al. 2017[4]
			SiKao (D5)	220	S1D5	KY982664	7°34'18.5"N<br>99°20'43.1"E	Manomat et al. 2017[4]
				58	S1D5	KY982676	7°34'18.5"N<br>99°20'43.1"E	Manomat et al. 2017[4]
			Nayong (D6)	226	S1D6	KY982658	7°33'42.5"N<br>99°41'41.8"E	Manomat et al. 2017[4]
			Yantakao (D7)	88603	S1D7	KY982661	7°23'09.1"N<br>99°40'02.2"E	Manomat et al. 2017[4]
			N/A	183	-	KY982663	N/A	Manomat et al. 2017[4]
				Reference	-	JX195640		Manomat et al. 2017[4]
L. martiniquensis								
	North (N)	Chiang Rai (1)	Phan (D2)	93	N1D2	OR135547	19°33'14.2"N<br>99°44'25.7"E	In this study
	South (S)	Trang (1)	Mueang (D1)	267615	S1D1	KY982641	7°33'28.7"N<br>99°36'35.1"E	Manomat et al. 2017[4]
				452157	S1D1	KY982642	7°33'28.7"N<br>99°36'35.1"E	Manomat et al. 2017[4]
				497	S1D1	KY982644	7°33'28.7"N<br>99°36'35.1"E	Manomat et al. 2017[4]
				662	S1D1	KY982645	7°33'28.7"N<br>99°36'35.1"E	Manomat et al. 2017[4]
				734	S1D1	KY982648	7°33'28.7"N<br>99°36'35.1"E	Manomat et al. 2017[4]
			Wang Wiset (D2)	422002	S1D2	OR135548	7°44'10.7"N<br>99°23'34.5"E	In this study
			Kantang (D3)	335A	S1D3	KY982640	7°24'19.9"N<br>99°30'53.9"E	Manomat et al. 2017[4]

Species	Region	Province	District	Isolate name	Location code	Accession no.	Coordinate (lat., long.)	Source
			Nayong (D6)	472123	S1D6	KY982643	7°33'42.5"N 99°41'41.8"E	Manomat et al. 2017[4]
			Hat Samran (D9)	816	S1D9	KY982651	7°14'26.6"N 99°34'36.2"E	Manomat et al. 2017[4]
			Ratsada (D10)	725	S1D10	KY982647	7°58'29.3"N 99°37'59.5"E	Manomat et al. 2017[4]
		Krabi (2)	Mueang (D1)	761	S2D1	KY982649	8°03'44.1"N 98°55'06.6"E	Manomat et al. 2017[4]
			Nuea Khlong (D3)	453	S2D3	KY982639	8°04'29.6"N 99°00'13.2"E	Manomat et al. 2017[4]
		Phuket (4)	Kathu (D1)	770605	S4D1	KY982650	7°54'32.7"N 98°20'00.4"E	Manomat et al. 2017[4]
			N/A	724	-	KY982646		Manomat et al. 2017[4]
				Reference	-	EF200012		Manomat et al. 2017[4]
L. donovani complex								
	South (S)	Trang (1)	Mueang (D1)	492733	S1D1	OR135549	7°33'28.7"N 99°36'35.1"E	In this study
				211377	S1D1	KY982629	7°33'28.7"N 99°36'35.1"E	Manomat et al. 2017[4]
				694	S1D1	KY982630	7°33'28.7"N 99°36'35.1"E	Manomat et al. 2017[4]
				239	S1D1	KY982632	7°33'28.7"N 99°36'35.1"E	Manomat et al. 2017[4]
				275629	S1D1	KY982633	7°33'28.7"N 99°36'35.1"E	Manomat et al. 2017[4]
				420	S1D1	KY982635	7°33'28.7"N 99°36'35.1"E	Manomat et al. 2017[4]
			Wang Wiset (D2)	321422	S1D2	KY982637	7°44'10.7"N 99°23'34.5"E	Manomat et al. 2017[4]
			Kantang (D3)	124	S1D3	OR135550	7°24'19.9"N 99°30'53.9"E	In this study
				612711	S1D3	OR135551	7°24'19.9"N 99°30'53.9"E	In this study
				742271	S1D3	OR135552	7°24'19.9"N 99°30'53.9"E	In this study
			Nayong Nuea (D4)	140118	S1D4	KY982631	7°33'51.5"N 99°41'41.7"E	Manomat et al. 2017[4]
		Krabi (2)	Koh Lanta (D2)	516016	S2D2	KY982636	7°39'26.5"N 99°02'28.6"E	Manomat et al. 2017[4]
		Nakhon Si Thammarat (3)	Na Bon (D1)	287804	S3D1	KY982634	8°15'44.0"N 99°35'46.1"E	Manomat et al. 2017[4]
			N/A	524184	-	KY982638		Manomat et al. 2017[4]

Species	Region	Province	District	Isolate name	Location code	Accession no.	Coordinate (lat., long.)	Source
				Reference	-	AJ000303		Manomat et al. 2017[4]
L. lainsoni								
	South (S)	Trang (1)	Mueang (D1)	66566A	S1D1	KY982654	7°33'28.7"N 99°36'35.1"E	Manomat et al. 2017[4]
				13840	S1D1	KY982655	7°33'28.7"N 99°36'35.1"E	Manomat et al. 2017[4]
			Kantang (D3)	320261	S1D3	OR135553	7°24'19.9"N 99°30'53.9"E	In this study
				75468	S1D3	KY982657	7°24'19.9"N 99°30'53.9"E	Manomat et al. 2017[4]
			Palien (D8)	723257	S1D8	KY982656	7°10'21.8"N 99°41'08.9"E	Manomat et al. 2017[4]
				Reference	-	FN398154		Manomat et al. 2017[4]
L. major								
	South (S)	N/A	N/A	426960	-	KY982652		Manomat et al. 2017[4]
				Reference		AJ000310		Manomat et al. 2017[4]
L. braziliensis								
				Reference		AJ300483		Manomat et al. 2017[4]
L. panamensis								
				Reference		AJ000298		Manomat et al. 2017[4]
L. guyanensis								
				Reference		AJ000300		Manomat et al. 2017[4]
L. tropica								
				Reference		AJ000301		Manomat et al. 2017[4]
L. aethiopica								
				Reference		EU683620		Manomat et al. 2017[4]
L. adleri								
				Reference		AJ300480		Manomat et al. 2017[4]
L. turanica								
				Reference		AJ000307		Manomat et al. 2017[4]
L. gerbilli								
				Reference		AJ300486		Manomat et al. 2017[4]
L. mexicana								
				Reference		AJ000312		Manomat et al. 2017[4]
L. infantum								
				Reference		AJ000303		Manomat et al. 2017[4]

Table S1. The geographic origin and haplotypes of 65 *Leishmania* species isolates in patients with HIV with reference *Leishmania* sequences used in this study. N/A: not applicable.
